# Decreased expression of dual-specificity phosphatase 9 is associated with poor prognosis in clear cell renal cell carcinoma

**DOI:** 10.1186/1471-2407-11-413

**Published:** 2011-09-26

**Authors:** Song Wu, Yong Wang, Liang Sun, Zhiling Zhang, Zhimao Jiang, Zike Qin, Hui Han, Zhuowei Liu, Xianxin Li, Aifa Tang, Yaoting Gui, Zhiming Cai, Fangjian Zhou

**Affiliations:** 1 Shenzhen Second People's Hospital, The First Affiliated Hospital of Shenzhen University, Shenzhen 518035, PR China; 2 Institute of Urology, Shenzhen PKU-HKUST Medical Center, Shenzhen 518036, Guangdong, PR China; 3 Anhui Medical University, Hefei 230022, An Hui, PR China; 4 Department of Urology, Sun Yat-Sen University Cancer Center, Guangzhou 510060, Guangdong, PR China

## Abstract

**Background:**

The molecular mechanisms involved in the development and progression of clear cell renal cell carcinomas (ccRCCs) are poorly understood. The objective of this study was to analyze the expression of dual-specificity phosphatase 9 (DUSP-9) and determine its clinical significance in human ccRCCs.

**Methods:**

The expression of *DUSP-9 *mRNA was determined in 46 paired samples of ccRCCs and adjacent normal tissues by using real-time qPCR. The expression of the DUSP-9 was determined in 211 samples of ccRCCs and 107 paired samples of adjacent normal tissues by immunohistochemical analysis. Statistical analysis was performed to define the relationship between the expression of DUSP-9 and the clinical features of ccRCC.

**Results:**

The mRNA level of *DUSP-9*, which was determined by real-time RT-PCR, was found to be significantly lower in tumorous tissues than in the adjacent non-tumorous tissues (p < 0.001). An immunohistochemical analysis of 107 paired tissue specimens showed that the DUSP-9 expression was lower in tumorous tissues than in the adjacent non-tumorous tissues (p < 0.001). Moreover, there was a significant correlation between the DUSP-9 expression in ccRCCs and gender (p = 0.031), tumor size (p = 0.001), pathologic stage (p = 0.001), Fuhrman grade (p = 0.002), T stage (p = 0.001), N classification (p = 0.012), metastasis (p = 0.005), and recurrence (p < 0.001). Patients with lower DUSP-9 expression had shorter overall survival time than those with higher DUSP-9 expression (p < 0.001). Multivariate analysis indicated that low expression of the DUSP-9 was an independent predictor for poor survival of ccRCC patients.

**Conclusion:**

To our knowledge, this is the first study that determines the relationship between DUSP-9 expression and prognosis in ccRCC. We found that decreased expression of DUSP-9 is associated with poor prognosis in ccRCC. DUSP-9 may represent a novel and useful prognostic marker for ccRCC.

## Background

Clear cell renal cell carcinoma is a common urological malignancy worldwide [1]. Although there have been immense improvements in the treatment of ccRCC during recent years, there is a gradual increase in the incidence of this disease. ccRCC initially presents as metastasis in 30% of patients, and up to 40% patients undergoing nephrectomy develop local recurrence or metastatic disease [2]. Although some environmental and genetic factors have been found to be associated with ccRCC, the molecular mechanisms involved in the initiation and progression of ccRCC are still unclear [3].

Dual-specificity phosphatase 9 (DUSP-9) is a member of the dual-specificity protein phosphatase subfamily [4,5]. DUSP-9 negatively regulates members of the mitogen-activated protein (MAP) kinase superfamily (e.g., ERK, JNK, p38), which are associated with cellular proliferation and differentiation [6,7]. Massively parallel sequencing studies have revealed the down-regulation of DUSP-9 in ccRCC [8]. However, since there is no published report on this phenomenon, the relationship between the expression of DUSP-9 and clinical significance needs to be clarified. In this study, we aimed to explore the expression of DUSP-9 and its clinical significance in ccRCC.

## Methods

### Patients and tissue specimens

Written informed consent was obtained from all patients, and the study was approved by the institutional review board of Sun Yat-sen University. For real-time RT-PCR analysis, we collected 46 paired samples of ccRCCs and adjacent normal tissues from patients who underwent radical nephrectomy between February 2008 and December 2009. The 46 patients included 40 men and 6 women with a median age of 50 years (range, 37-75 years). The fresh tissues were immediately immersed in RNAlater (Qiagen; Germany) after surgical resection, stored at 4°C overnight to allow thorough penetration of the tissue, and then frozen at -80°C. In addition, we performed an immunohistochemical assay of 211 paraffin-embedded samples of ccRCCs and 107 adjacent normal renal tissue samples collected from patients between 1999 through 2007. The characteristics of these 211 patients are listed in Table 1. None of the patients underwent radiotherapy or chemotherapy before surgery. The histological and clinical diagnosis of the tumors in all these patients was performed by the Cancer Center of Sun Yat-sen University. The disease stage of each patient was classified or reclassified according to the 2002 American Joint Committee on Cancer (AJCC) staging system [9].

**Table 1:** Correlation between DUSP-9 expression and clinical pathologic features of the patients with clear cell renal cell carcinoma

Clinical-pathologic variables	n	DUSP-9 expression		χ2	p
				
		Low	High		
**All cases**	**211**	**117**	**94**	**4.667**	**0.031**
**Male**	**140**	**85**	**55**		
**Female**	**71**	**32**	**39**		
**Age (yrs)**				**0.417**	**0.518**
> **50**	**104**	**60**	**44**		
≤ **50**	**107**	**57**	**50**		
**Pathologic stage**				**17.112**	**0.001**
**I**	**128**	**58**	**70**		
**II**	**23**	**13**	**10**		
**III**	**31**	**22**	**9**		
**IV**	**29**	**24**	**5**		
**Fuhrman Grade**				**14.492**	**0.002**
**I**	**39**	**28**	**11**		
**II**	**123**	**72**	**51**		
**III**	**34**	**10**	**24**		
**IV**	**15**	**7**	**8**		
**Tumor size (cm)**				**10.906**	**0.001**
**≤7**	**136**	**64**	**72**		
**>7**	**75**	**53**	**22**		
**T stage**				**14.914**	**0.001**
**T_1_**	**135**	**63**	**72**		
**T_2_**	**35**	**21**	**14**		
**T_3_, T_4_**	**41**	**33**	**8**		
**N stage**				**6.248**	**0.012**
**N0**	**184**	**96**	**88**		
**N+**	**27**	**21**	**6**		
**Metastasis**				**7.708**	**0.005**
**No**	**188**	**98**	**90**		
**Yes**	**23**	**19**	**4**		
**Recurrence**				**24.050**	**<0.001**
**No**	**181**	**88**	**93**		
**Yes**	**30**	**29**	**1**		

### Real-Time qPCR

Total RNA was extracted using the TRIzol solution (Invitrogen; Carlsbad, CA) according to the manufacturer's protocol; RNase-free DNase I was used to remove the DNA contamination. M-MLV reverse transcriptase (Fermentas; American) was used according to the manufacturer's recommendations to treat 2 μg of the total RNA for synthesizing the ?rst-strand cDNA. The cDNA was then subjected to real-time quantitative PCR for evaluation of the relative mRNA levels of DUSP-9 and GAPDH (as an internal control) with

the corresponding primer pairs ( DUSP-9 sense strand: 5'-TATGCCACGCCCTTTGAG-3', DUSP-9 antisense strand: 5'-CACAGCAGGATGTAGGAGATGA-3'; GAPDH sense strand: 5'-GCTCTCTGCTCCTCCTGTTC-3', GAPDH antisense strand: 5'-GACTCCGACCTTCACCTTCC-3'). Gene-speci?c ampli?cation was performed using an Applied Biosystems (ABI 7000) real-time PCR machine with a 20-μl PCR reaction mixture containing 1 μl of cDNA (synthesized as described above), 10-μl SYBR Green master mix (Invitrogen; Carlsbad, CA), and 40 nM of each pair of oligonucleotide primers. The ampli?cation conditions were 50°C (2 min) and 95°C (2 min) for 1 cycle and 95°C (15 sec), 55°C (30 sec), and 72°C (40 sec) for 40 cycles. Regression curves were calculated for each sample, and the relative amount of mRNA was calculated from the threshold cycles by using the software provided with the instrument (Version 17.0 SPSS Inc.). Relative expression levels of the target genes were normalized to the geometric mean of the internal control gene, GAPDH. The data was analyzed using the comparative threshold cycle (2^-ΔCT^) method.

### Immunohistochemical assay

An immunohistochemical assay was performed to examine DUSP-9 expression in the 211 ccRCC samples and 107 paired samples of adjacent normal renal tissue. All procedures were performed using classical protocols. In brief, paraffin-embedded specimens were cut into 5-μm sections and baked at 65°C for 30 min. The sections were deparaffinized with xylene and rehydrated, submerged into 0.01 M citrate buffer (pH 6.0) antigen retrieval buffer, and then microwaved for antigen retrieval. They were then treated with 3% hydrogen peroxide in methanol to quench the endogenous peroxidase activity, which was followed by incubation with 10% bovine serum albumin to block nonspecific binding. The DUSP-9 protein was detected by using a mouse monoclonal antibody against DUSP-9 (Abcam; Cambridge, MA, USA). The specimens were incubated overnight at 4°C with anti-DUSP-9 antibody (1:250). The negative control for immunohistochemical analysis was obtained by replacing the primary antibodies with an antibody diluent. After being washed in phosphate buffered saline (PBS), the sections were treated with MaxVision™ HRP-Polymer anti-Mouse IHC Kit (Maixin Bio; Fujian, China) at 37°C for 15-20 min. The tissue sections were immersed in 3-amino-9-ethyl carbazole, counterstained with Mayer's hematoxylin, dehydrated, and finally mounted in Crystal Mount.

The formalin-fixed, paraffin-embedded sections were reviewed for the degree of immunostaining and scored by 2 independent observers. The proportion of cells expressing DUSP-9 varied from 0% to 100%, and the intensity of staining varied from weak to strong. The proportion of DUSP-9- expressing tumor cells was scored as follows: 0, no positive cells; 1, <5%; 2, 6%-25%; 3, 26%-50%; 4, 51%-75%; and 5, >75% according to Tsuchiya et al. The staining intensity was graded according to the mean optical density [10-12]: 0, no staining; 1, weak staining (light yellow); 2, moderate staining (yellow brown); and 3, strong staining (brown). Staining index was calculated as the multiplication of staining intensity score and the proportion of DUSP-9-positive tumor cells. We evaluated DUSP-9 expression in benign kidney tissue and malignant lesions on the basis of the staining index values, with scores of 0, 1, 2, 3, 4, 5, 6, 8, 9, 10, 12, and 15. The cutoff values for DUSP-9 expression were chosen on the basis of a measure of heterogeneity in overall survival rates, which was calculated using the log-rank test. An optimal cutoff value was identified: a staining index score of ≥5 was considered as high DUSP-9 expression, whereas a staining index score of ≤4 was considered as low DUSP-9 expression.

### Statistical analysis

All statistical analysis was carried out with the SPSS 17.0 statistical software package. In the real-time RT-PCR and immunohistochemical assays, paired-sample *t *tests were used to analyze the significance of the differences in mRNA and protein expression between ccRCCs and the adjacent normal tissues. The χ^2^-test for proportion was used to analyze the relationship between DUSP-9 expression and clinical significance. Survival curves were plotted by the Kaplan-Meier method and compared by the log-rank test. We determined that the assumption of proportional hazards was met in all Cox regression models. The significance of various variables for survival was analyzed by the Cox proportional hazards model in multivariate analysis. p < 0.05 was considered to be statistically significant.

## Results

### Real-time quantitative RT-PCR analysis of DUSP-9 expression

**Figure 1:**
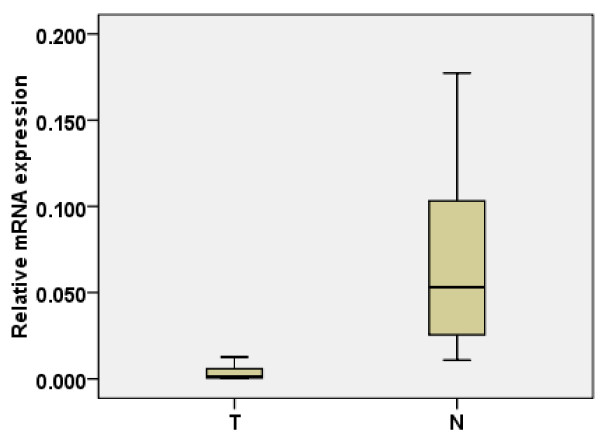
**Real-time quantitative RT-PCR analysis of *DUSP-9 *expression**. The relative expression of *DUSP-9 *mRNA in RCC tumor tissue samples was lower than that in the paired adjacent normal (N) tissue samples (n = 46, *P *< 0.001). The bottom and the top of the box represent the 25^th ^and the 75^th ^percentile, respectively, and the band near the middle of the box is the 50^th ^percentile (the median). The ends of the whiskers represent the 2.5^th ^percentile and the 97.5^th ^percentile.

The transcription level of *DUSP-9 *was determined by quantitative RT-PCR assays of 46 ccRCC tumor samples and the paired adjacent normal tissue samples. In 45 tumor samples, the mRNA level of *DUSP-9 *was significantly lower than that in the adjacent normal tissue sample (p < 0.001, paired-sample *t *tests, Figure 1).

### Immunohistochemical analysis of the expression of DUSP-9 protein in 107 paraffin-embedded ccRCC samples (T) and the paired adjacent normal renal tissue (N)

**Figure 2:**
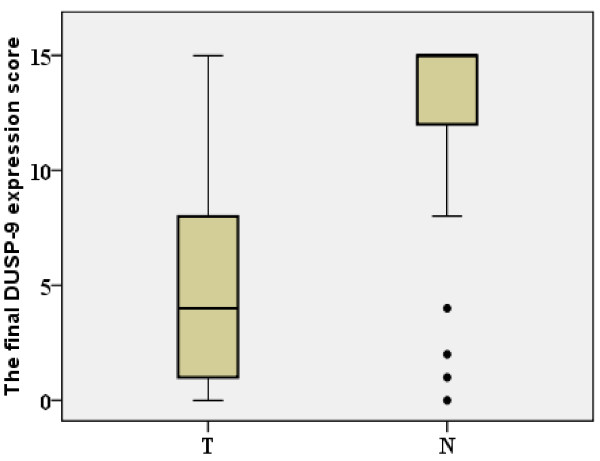
**Decreased protein expression of DUSP-9 in ccRCC**. The relative protein expression of DUSP-9 in ccRCC tumor (T) tissue samples was lower than that in the paired adjacent normal (N) tissue samples (n = 107, *P *< 0.001). The bottom and top of the box are the lower and upper quartiles, and the band near the middle of the box is the median. The ends of the whiskers represent the 2.5^th ^percentile and the 97.5^th ^percentile. Four black spots represent the special value outliers.

**Figure 3:**
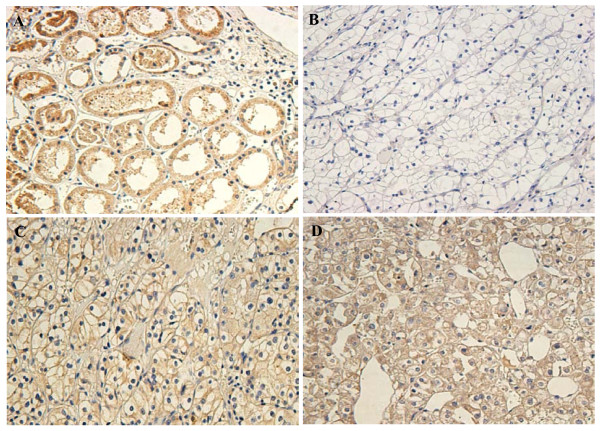
**Immunohistochemical analysis of the expression of DUSP-9 protein**. DUSP-9 is mainly localized within the nuclei and cytoplasmic. Immunostaining of the adjacent normal tissue samples(A) and the ccRCC tumor tissue samples(B) showed a sharp contrast between the negatively stained infiltrative tumorous area.(B): Negative or weak DUSP-9 staining in cancerous tissue (400×). (C): Moderate DUSP-9 staining in cancerous tissue(400×). (D): Strong DUSP-9 staining in most of tumor cells (400×).

Expression and subcellular localization of protein were determined by immunohistochemical analysis in 107 paraffin-embedded ccRCC tissues and 107 paired specimens of adjacent normal tissues (Figure 2). In normal renal tissue, specific DUSP-9 was localized mainly in the cytoplasm of renal cells in the form of yellow-brown granules (Figure 3A). The DUSP-9 protein expression in the 102 tumor tissue samples was lower than that in the adjacent normal tissue samples (p < 0.001, paired-sample *t *test, Figure 3B).

### Immunohistochemical analysis of DUSP-9 expression in 211 ccRCC samples (T) and its relationship with the clinical features

To further investigate the effect and the prognostic value of DUSP-9, immunohistochemical analysis was performed to assess the expression of DUSP-9 in 211 ccRCC tissue blocks. Overall, 117 of the 211 tumor samples showed low expression of DUSP-9(score ≤4), whereas 94 samples showed high expression (score ≥5). The correlation between the expression of DUSP-9 and various clinicopathological parameters are listed in Table 1. Intense expression of DUSP-9 in ccRCC samples was correlated with gender (p = 0.031), pathologic stage (p = 0.001), Fuhrman grade (p = 0.002), tumor size (p = 0.001), T stage (p = 0.001), N classification (p = 0.012), metastasis (p = 0.005), and recurrence (p < 0.001), but it was not correlated with age. Low expression of DUSP-9 was noted in 46.7%, 60.0%, and 80.5% of T1, T2, and T3/4 stage ccRCCs respectively (p = 0.001, χ2 test). Low expression of DUSP-9 was not observed in 47.1% and 70.7% of ccRCCs with size ≤7 cm and >7 cm respectively (p = 0.001, χ2 test). Low expression of DUSP-9 was seen in 52.2% and 77.8% of N0 and N1/2 stage ccRCCs respectively (p = 0.012, χ2 test). Low expression of DUSP-9 was seen in 52.1% and 82.6% of ccRCCs with or without metastasis respectively (p = 0.005, χ2 test). Low expression of DUSP-9 protein was seen in 48.6% and 96.6% of ccRCCs with or without recurrence respectively (p < 0.001, χ^2 ^test).

### Survival analysis

**Figure 4:**
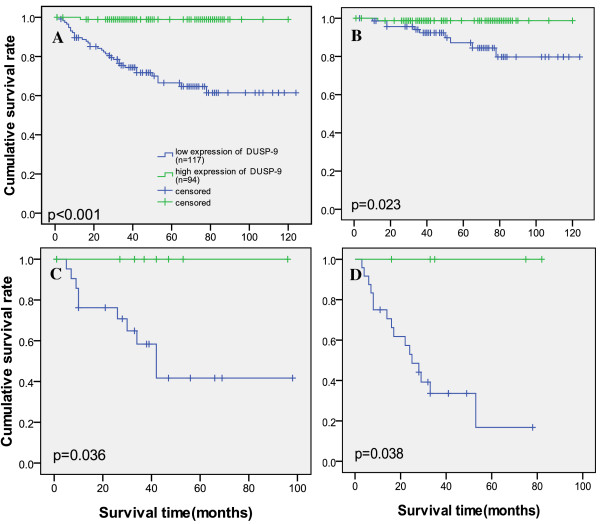
**Survival analysis of primary ccRCC patients (n = 211)**. (A) Overall survival. Kaplan-Meier survival analysis of primary ccRCC patients (n = 211) after surgical resection with low DUSP-9 expression (n = 117) and high DUSP-9 expression (n = 94). The survival rate for patients in the DUSP-9 low group was significantly lower than that for patients in the DUSP-9 positive group (log-rank test, p<0.001). (B) Pathological stage I-II; (C) Pathological stage III; (D) Pathological stage IV. Statistical analysis of the difference between DUSP-9 high-expressing and low-expressing tumors was compared in the I-II( B), III(C) and IV (D) patient subgroups. The longest follow-up time is 124 months.

Kaplan-Meier analysis and the log-rank test were used to calculate the effect of the DUSP-9 expression on survival. The 5-year survival in the group of patients with high DUSP-9 expression was 97%, but it was 62.1% in the group of patients with low DUSP-9 expression (Figure 4A). The log-rank test showed that survival rates were significantly different between these 2 groups (p < 0.001). Furthermore, the relationship of DUSP-9 expression with prognosis was determined in 211 patients, which were divided into 3 subgroups depending on the pathologic stage. Patients with tumors exhibiting high DUSP-9 expression had significantly longer overall survival than those with low expression of DUSP-9 either in the stage I plus II subgroup (n = 151; log-rank, p = 0.023; Figure 4B), the stage III sub group (n = 31; log-rank, p = 0.036; Figure 4C), or the stage IV subgroup (n = 29; log-rank, p = 0.038; Figure 4D). Patients with tumors high DUSP-9 expression had significantly longer overall survival than those with low expression of DUSP-9 either in the Fuhrman grade I subgroup(n = 39; log-rank, p = 0.005; Additional file 1 Figure S1A), II subgroup (n = 123; log-rank, p < 0.0001; Additional file 1 Figure S1B), the stage III sub group (n = 34; log-rank, p < 0.001; Additional file 1 Figure S1C), or the stage IV subgroup (n = 15; log-rank, p = 0.019; Additional file 1 Figure S1D).

Univariate Cox regression analysis showed that tumor size, T stage, N stage, metastasis, Fuhrman grade and DUSP-9 expression were significantly associated with overall survival (Table 2). Furthermore, multivariate Cox regression analysis revealed that only DUSP-9 expression and Fuhrman grade were independent predictors for the overall survival of ccRCC patients (p = 0.005, respectively; Table 2), whereas the others factors were not independently related to the survival of ccRCC patients.

**Table 2:** Cox Regression analysis of the overall survival rates associated with different prognostic variables in patients with ccRCC

Variables	Univariate analysis		Multivariate analysis	
	
	Hazard ratios (95% confidence interval)	p	Hazard ratios (95% confidence interval)	p
**Tumor size**	**(3.339-15.178)**	**<0.001**	**(0.088-3.118)**	**0.478**
**T stage**	**(2.177-4.177 )**	**<0.001**	**(0.629-5.052)**	**0.276**
**N stage**	**(1.389-4.379 )**	**= 0.002**	**(0.438-3.119)**	**0.755**
**Metastasis**	**(2.705-11.837 )**	**<0.001**	**(0.100-6.355)**	**0.832**
**Fuhrman Grade**	**(35.53-630.43)**	**<0.001**	**(27.23-569.2)**	**<0.001**
**DUSP-9**	**(0.004-0.229 )**	**0.001**	**(0.088-3.118)**	**0.008**

## Discussion

Clear cell renal cell carcinoma accounts for about 2% of all cases of cancers, with an annual increase of 1.5-5.9% worldwide [13,14]. The initial treatment is usually a radical or partial nephrectomy, which remains the mainstay of curative treatment [15]. Unfortunately, ccRCC is resistant to radiation therapy and chemotherapy, but some tumors respond to molecular-targeted therapy. Therefore, identification of specific molecular biomarkers of ccRCC is an essential prerequisite. Although the numerous molecular markers, such as p53, vascular endothelial growth factor (VEGF), hypoxia inducible factor, Ki67 (proliferation), have been investigated as prognostic variables in ccRCC, the molecular mechanisms of the initiation and progression of ccRCC still remain unclear [16,17]. Massively parallel sequencing analysis showed that *DUSP-9 *is downregulated in ccRCC [9].

DUSP-9 is a member of the dual-specificity protein phosphatase subfamily and is expressed only in the placenta, kidney, and during the fetal life. Moreover, DUSP-9 is known to be associated with squamous cell carcinoma (SCC) and can independently induce SCC [5]. DUSP-9 inactivates the target kinases of squamous carcinoma cells by dephosphorylating both the phosphoserine/threonine and phosphotyrosine residues. DUSP-9 negatively regulates members of the mitogen-activated protein (MAP) kinase superfamily (MAPK, p38, SAPK), which are associated with cellular proliferation and differentiation [18-20]. Molly Kulesz-Martin et al. found that DUSP-9 reconstitution resulted in G2-M-associated cell death and microtubule disruption. Loss of DUSP-9 was associated with SCC, and it independently induced SCCs relative to benign tumors in mouse skin. Reconstitution of DUSP-9 expression in malignant tumor cells induces cell death and tumor suppression [6,21,22].

However, to our knowledge, the key feature of this study is that this is the first study to report the clinical significance of DUSP-9 in ccRCC. This is also the first study aimed at evaluating the possibility of using DUSP-9 as a clinically potential indicator for disease progression, as well as a prognostic marker for patient survival in tumors.

In this study, we showed that *DUSP-9 *mRNA and protein expression were significantly different between the ccRCC and the adjacent normal renal tissue samples. Furthermore, immunohistochemical analysis showed that DUSP-9 expression was moderate to low in ccRCCs, while it was high in the adjacent normal tissues. Accordingly, we found that DUSP-9 expression was reduced in a large number of human clinical ccRCC samples. The decreased expression of DUSP-9 was correlated with gender, pathologic stage, Fuhrman grade, tumor size, recurrence, TNM stage, and prognosis. Patients with lower DUSP-9 expression had shorter survival time, and those with higher DUSP-9 expression had a longer survival time. In addition, the relationship of DUSP-9 expression with prognosis was determined in the patients, which were divided into 3 subgroups depending on the pathologic stage. We found that DUSP-9 could be a valuable prognostic marker for ccRCC patients at all disease stages. Consistent with previous reports of other cancers, low-expression of DUSP-9 indicated poor prognosis for patients with ccRCC.

DUSP-9 expression is correlated with low Fuhrman grade. This result did not match with the other correlations. However, in the survival analysis, we found that patients with tumors high DUSP-9 expression had significantly longer overall survival than those with low expression of DUSP-9 either in the Fuhrman grade I subgroup, II subgroup, the stage III sub group, or the stage IV subgroup. We observed that there are more cases with low Fuhrman grade. In addition, this was a single hospital-based, retrospective study. In addition to this observation, we have, in particular, found that DUSP-9 expression is correlated with low Fuhrman grade.

The TNM stage of ccRCC and Fuhrman grade are closely related to its prognosis [23-25]. In our study, the results of univariate Cox regression analysis showed that tumor size, T stage, N stage, metastasis, Fuhrman grade and DUSP-9 expression were significantly associated with overall survival. Furthermore, multivariate Cox regression analysis revealed that only DUSP-9 expression and Fuhrman grade were independent predictors for the overall survival of ccRCC patients. Thus, our findings indicate that the DUSP-9 expression level has a significant correlation with clinicopathological features and is a potential prognostic marker for ccRCC.

Our study was a single hospital-based, retrospective study. It should be pointed out that unmeasured differences may exist and may distort the study results. A multicenter or community-based prospective study with more extensive collection of potential confounders is required. In addition, the correlation of DUSP-9 with the above-mentioned molecular markers needs to be investigated further. Apparently, more studies are required to explore the relationship between the DUSP-9 gene and other genes such as p38 that may be associated with ccRCC.

## Conclusion

In summary, we demonstrated the down-regulation of DUSP-9 in ccRCC and its correlation with poor prognosis by using a large number of clinical samples. Our results indicate the role of DUSP-9 as a prognostic factor and a potential tumor suppressor in primary ccRCC. Measurement of DUSP-9 expression in primary ccRCC can help stratify the patients for prognosis. Furthermore, DUSP-9 may be a new potential therapeutic target for ccRCC in the future.

## Competing interests

The authors declare that they have no competing interests.

## Authors' contributions

SW, YW, LS, ZLZ were responsible for data collection and analysis, experiment job, interpretation of the results, and writing the manuscript. HH, ZKQ, YTG, XXL, AFT, ZWL and MZJ were responsible for conducting the data analysis, reviewing and scoring the degree of immunostaining of sections in cooperation with LS. FJZ and ZMC were responsible for experimental design, analysis and interpretation. All authors have read and approved the final manuscript.

## Pre-publication history

The pre-publication history for this paper can be accessed here:

http://www.biomedcentral.com/1471-2407/11/413/prepub

## Supplementary Material

Additional file 1 **Additional file 1, Figure S1**. Survival analysis of difference Fuhrman grade. Click here for file
