# Supplementary material for: Decreased expression of dual-specificity phosphatase 9 is associated with poor prognosis in clear cell renal cell carcinoma
Source: BMC Cancer. 2011 Sep 26;11:413. doi: 10.1186/1471-2407-11-413 (PMC3198720; doi:10.1186/1471-2407-11-413)
Supplement: Additional file 1 — , Figure S1. Survival analysis of difference Fuhrman grade. [file 1471-2407-11-413-S1.DOC]

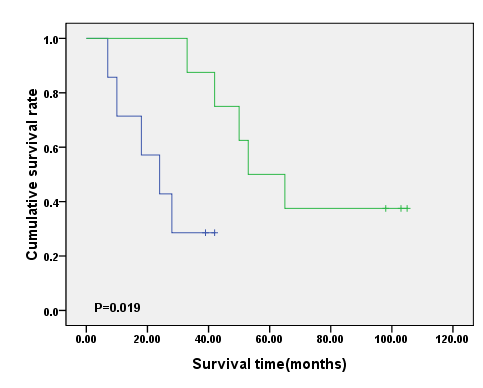

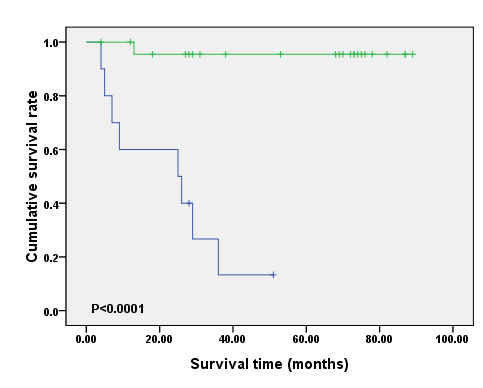

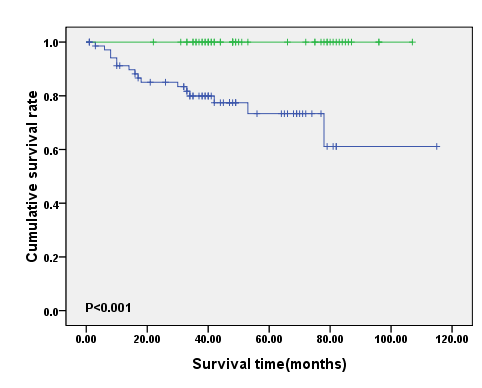

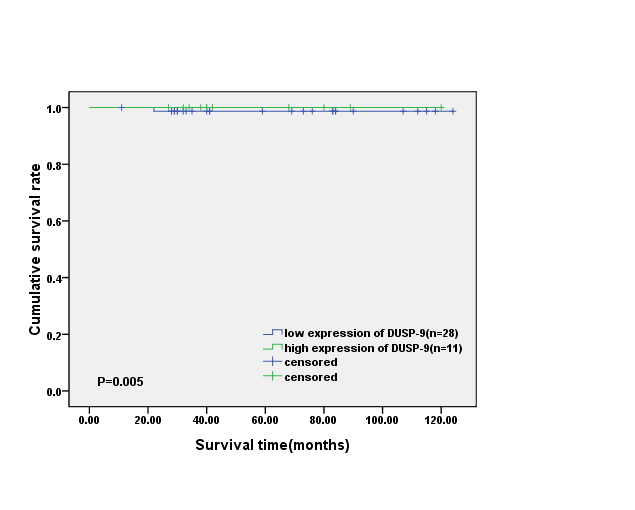


**C**

**A**

**B**

**D**

Figure S1. Survival analysis of difference Fuhrman grade.

Patients with tumors high DUSP-9 expression had significantly longer overall survival than those with low expression of DUSP-9 either in the Fuhrman grade I subgroup(n = 39; log-rank, p = 0.005; A) , II subgroup (n = 123; log-rank, p < 0.0001; B), the stage III sub group (n = 34; log-rank, p < 0.001; C), or the stage IV subgroup (n = 15; log-rank, p = 0.019; D).
